# The Structure of Morpho-Functional Conditions Determining the Level of Sports Performance of Young Badminton Players

**DOI:** 10.1515/hukin-2015-0077

**Published:** 2015-10-14

**Authors:** Janusz Jaworski, Michał Żak

**Affiliations:** 1 Department of Physical Education, Institute of Sport, Department of Sport and Kinesiology, University School of Physical Education in Krakow.; 2 Department of Physical Education, Institute of Sport, Section of Athletics Sports and Recreational Training, University School of Physical Education in Krakow.

**Keywords:** badminton, morpho-functional profile, selection, training

## Abstract

The aim of the study was to determine the structure of morpho-functional models that determine the level of sports performance in three consecutive stages of training of young badminton players. In the course of the study, 3 groups of young badminton players were examined: 40 preadolescents aged 11–13, 32 adolescents aged 14–16, and 24 adolescents aged 17–19. The scope of the study involved basic anthropometric measurements, computer tests analysing motor coordination abilities, motor skills encompassing speed, muscular power and strength, and cardiorespiratory endurance. Results of the study indicate that the structure of morpho-functional models varies at different stages of sports training. Sets of variables determining sports performance create characteristic complexes of variables that do not constitute permanent models. The dominance of somatic features and coordination abilities in the early stages of badminton training changes for the benefit of speed and strength abilities.

## Introduction

Badminton is a very popular racquet sport among people of all ages and numerous nationalities, and its popularity is growing steadily (Sakurai and Ohtsuki, 2000). Every movement sequence that can be observed during badminton game typically requires a set of predispositions, in other words, a certain amount of power, speed, endurance and coordination. Nevertheless, even a preliminary observation is sufficient to describe the physical effort of a badminton player as dominated by speed and strength abilities. On the one hand, it entails anaerobic loads (maximal power output in a very short period of time), namely starts, changes in the direction of movements, fast and strong shuttle strokes, jumps, etc., and on the other hand, it involves aerobic loads resulting from the duration of the game, and a number of movement sequences repeated in different configurations. However, so far very few studies have analyzed the game time structure taking into account the total match duration and active game time. As Cabello et al. (1997) demonstrated, the average of total match duration (SD) was 32 min 52 s (±15 min 2 s), and the total work time was 9 min 4 s (±3 min 1 s). It has also been demonstrated that these times increase along with the competition level. For instance, during the Olympic Games in 1996, the total mean game duration amounted to 55 min and the work time to 25 min 26 s (Cabello et al., 2000). Conversely, the literature abounds with studies on physiological aspects of players’ preparation (Andersen et al., 2007; Ghosh, 2008; Hughes and Cosgrovee, 2007; Kim et al., 2002; Ooi et al., 2009, Wonisch et al., 2003). Developing coordination abilities and their influence on specific skills and game performance seem to be an important problem in the theory of badminton sports training. In this research stream, the studies conducted by Ando et al. (2002), Chin et al. (1995), Mooney and Mutrie (2000), Sakurai and Ohtsuki (2000), Wang et al. (2008), Wang et al. (2009), Yuan et al. (1995), deserve particular attention. From the practical point of view, the analyses conducted in order to establish the level of basic somatic features that affect performance of badminton players seem useful (Berral de la Rosa et al., Campos et al., 2009; Chang et al., 2006; 2010; Heller, 2010). Recently, another research analysing these issues in the context of the dominant and non-dominant body side was published (Abián et al., 2012). However, a low correlation observed occasionally between the study results and players’ rating lists (r = 0.65) can indicate the presence of other important factors that are indispensable for achieving success in badminton at the elite level (Chin et al., 1995). Among these factors, psychological characteristics of a badminton player play an important role (Zivdar et al., 2012).

As demonstrated above, despite a high level of complication and complexity of body movements coupled with occasional extreme exertion, in Poland this relatively young Olympic discipline has rarely been the subject of comprehensive scientific research. The seemingly large number of studies are reflected by the heuristics concerning mainly contributory studies. These investigations have examined only selected motor aspects, usually with the study groups that consisted of already developed adult players. Far less attention has been paid to analyses taking into account the development of players, their predispositions and skills at particular stages of training, as well as a comprehensive assessment of morphological conditions and functional sports performance.

Considering the aforementioned observations, the fundamental aim of this study was to establish the structural and functional determinants of the level of sports performance in three consecutive stages of training of young badminton players. The study attempted to answer the following research questions:

1. What is the structure of morpho-functional models of young badminton players at different stages of training?
2. Is the set of variables determining sports performance in badminton stable, or does it change with both the age of subjects and their sports level?

## Material and Methods

This study comprised results collected in 2009 among 96 badminton players. A total of 40 boys aged 11–13 were registered as preadolescents, 32 as adolescents aged 14–16, and 24 as adolescents aged 17–19. The average competition experience in the preadolescent group equalled 3.8 years; in the ages 14–16 adolescent category, 5.9 years; and in the group of 17–19 adolescents, 8.2 years. The level of sports performance of the examined players was established on the basis of classification lists prepared by the Polish Badminton Association at the end of the season and supplemented with the “experts” method. The study material included the results achieved by players in the adolescent group aged 14–16 and junior Polish national teams (including three top-ranked players from each group), and champions of individual macro regions in the preadolescent category. Great care was taken that each sports category encompassed an equal number of subjects placed in the consecutive tenths of the ranking (players from the first 50–60 places of the ranking were considered).

### Measurements of somatic variables

A standard Martin’s method was used for measurements of morphological variables such as body height, upper limb length, sitting body height, subischial leg length, length of the arm with the racket for a forehand grip, shoulder and hip width, body mass and its components (LBM and fat mass were evaluated using a TANITA TBF-551 body composition analyser), flexibility (Eurofit 1993) and wrist flexibility.

The analysis also included selected fitness-related abilities such as: a standing long jump (lower limb MAP), static strength measured with a handgrip dynamometer, dynamic strength of the abdominal muscles, a 10 × 5 m shuttle run, a “beep test” – cardiorespiratory endurance (all the tests were performed according to the methodology of the Eurofit 1993 test battery), an overhead 2 kg medicine ball throw (upper limb MAP), measurement of muscular force differentiation ability using a handgrip dynamometer (difference between the measurement of the maximum and indicated force), a run with changes in directions (an “envelope-shaped” run, with total time of three repetitions recorded), MAP tests – an overhead 1 kg medicine ball throw from a kneeling position, a shuttle run 10 × 3 metres and tapping with the 2 kg medicine ball (10 cycles of tapping with the ball held with both hands over the head against the wall and the ground between the widespread lower limbs) – the results of both tests were expressed in s.

With regard to contemporary research tendencies in the field of measurement of coordination-related aptitudes, we used computer tests of coordination motor abilities that evaluated kinaesthetic differentiation of temporal movement parameters, movement frequency, visual-motor coordination (an optional mode and a forced mode), spatial orientation (an optional mode and a forced mode), auditory reaction time (minimal, mean, maximal), visual reaction time (minimal, mean, maximal), selective reaction time to visual and auditory stimuli (minimal, mean, maximal), rhythmization, coupled motions, kinaesthetic differentiation ability (spatial and dynamic parameters). The same person conducted all tests using a special program for assessing coordination abilities. The tests were carried out by means of a Toshiba R15 laptop with the Windows XP Tablet OS. Before each test, a trial test was conducted. The trial ended when the test supervisor decided that the participant had understood and performed the stated tasks according to the testing instructions. The tests took place in a separate room that ensured quiet and allowed the participant to focus on the tasks. Computer tests were conducted in the afternoon, between 3 p.m. and 6 p.m., at the same time of day for a given participant. Due to the possible effect of fatigue on performance in coordination ability tests, coordination tests were conducted first, followed by fitness ability tests.

Research procedures were approved by the Bioethical Committee at the Regional Medical Chamber in Cracow (approval No. 51/KBL/OIL/2010).

### Statistical Analysis

In this study, the following statistical methods were applied:

1. Based on factor analysis, a reduction in the number of variables included in the study was performed. A variant of factor analysis based on the Hotelling’s principal component method, modified by Tucker, and supplemented by the Varimax rotation was applied.
2. In order to determine the combined influence of variables selected during the factor analysis on the level of sports performance, a stepwise regression model was employed. The multiple determination model applied estimated the combined effect of significant parameters that were selected from those included in the analysis (26 variables that had the strongest impact on sports performance). Multiple correlation was run on the measurement results obtained in three separate sports groups (preadolescents aged 11–13, adolescents aged 14–16, and adolescents aged 17–19). Considering the subject of the present study, the results of both analyses were not presented in this manuscript. Nevertheless, they were utilized for further detailed analyses.
3. Next, morpho-functional profiles were calculated in training groups by employing mean normalised results and standard deviations of the entire population of examined badminton players (a T scale). Since correlations between multiple traits and abilities with a morphological age and considerably lower dispersion of results in the sets of morphological age were found, the normalisation performed was related to this particular area.

## Results

The present study shows a detailed dependency analysis involving developmental aspects and the comparison of variable values (previously specified on the basis of factor analysis) that comprise individual models. To this end, for each category of badminton players, relevant morpho-functional profiles taking into account their morphological age were created. Appropriate models were presented in a graphical form and, additionally, against average values calculated for 10 and 19-year-old players, as well as mean values for all variables that comprised the model. In a sense, such an approach allowed comparisons of different possibilities of training of selected variables, their level at the recruitment stage and the current degree of developmental progression to be made. Table 1 presents the structure of morpho-functional models in the T scale for particular age groups. The issues that were considered are in fact answers to the questions related to morpho-functional models in badminton and their age-dependent structure, and provided the means to particularise them. The present study enabled subtle differences between individual parameters in relation to their contribution to the building of the described models to be captured.

The comparative analysis of all averaged modules allowed their variability to be established at a level of 1.63 of the standard deviation. However, it is worth remembering that the variables that comprised these modules were characterised by great differences in variability ranges in the entire population of examined badminton players.

**Figure 1:**
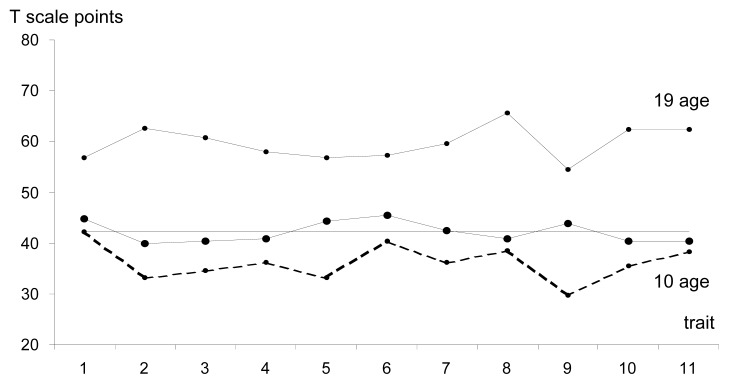
The profile of variables of the morpho-functional model in the preadolescent group in morphological age categories 1. Wrist flexibility, 2. Length of the arm with a racquet, 3. Shoulder width, 4. Spatial orientation, 5. Mean visual reaction time, 6. Coupled motions, 7. Running speed 8. Lower limb MAP, 9. Cardiorespiratory endurance, 10. Upper limb MAP 11. Static strength

In the preadolescent group, the inter-trait differentiation ranged within 0.55 of standard deviation (Figure 1, Table 1). Above the module, the following parameters were located: coupled motions, wrist flexibility, mean visual reaction time and cardiorespiratory endurance. It needs to be noted, however, that all these characteristics analysed in parallel with physical abilities indicated considerably less chance for further development, especially in relation to Maximal Anaerobic Power of lower and upper limbs. In the preadolescent group, a fairly balanced profile of characteristics and motor skills can be observed. In this case, it is difficult to use the term “model”, as in this set a strong influence of natural selection can be observed. However, it is safe to assume that although at the recruitment phase the aforementioned coordination abilities are essential, the level of sports performance (or, rather, the effectiveness of a badminton player at this stage of training) is determined by a number of other factors, predominantly physical characteristics and wrist flexibility.

**Figure 2:**
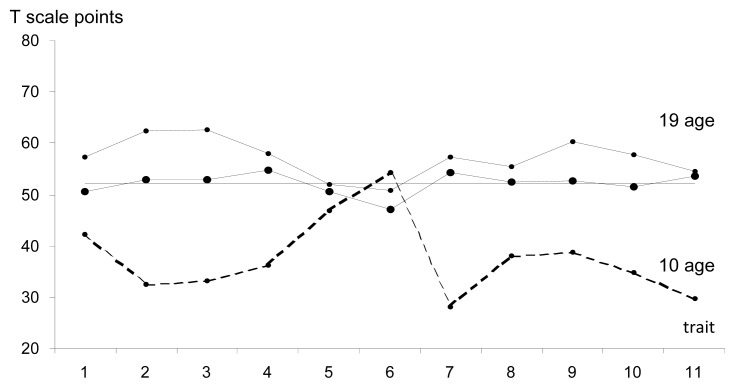
The profile of variables of the morpho-functional model in the adolescent group aged 14–16 in morphological age categories 4. Agility, 2. Body height, 3. Length of the arm with a racquet 4. Spatial orientation, 5. Rhythmisation, 6. Kinaesthetic differentiation 7. Visual-motor coordination, 8. Movement frequency, 9. Running speed 10. Abdominal muscle strength, 11. Cardiorespiratory endurance

In the group of adolescents aged 14–16 (Figure 2, Table 1), a slightly sharper differentiation in the contribution of individual parameters to shaping of the level of sports performance could be observed. However, it needs to be noted that this was determined by a relatively low score in kinaesthetic differentiation capabilities. Other variables were close to the mean value, while the dominant abilities seemed to include spatial orientation and visual-motor coordination. With respect to somatic traits (e.g., body height and the length of the arm) and physical abilities (e.g., cardiorespiratory endurance, abdominal muscle strength, running speed evaluated with a zigzag test), they are characterized by earlier development, which is shorter in later stages of training. Similar observation can be made for other coordination abilities, such as kinaesthetic differentiation, rhythmisation and frequency of movements.

The authors put forward a thesis that in the adolescent group aged 14–16, the level of sports performance was determined to some extent by physical development, predominantly versatile fitness coupled with distinct dominance of coordination abilities, mainly due to the number of its components that were revealed.

**Figure 3:**
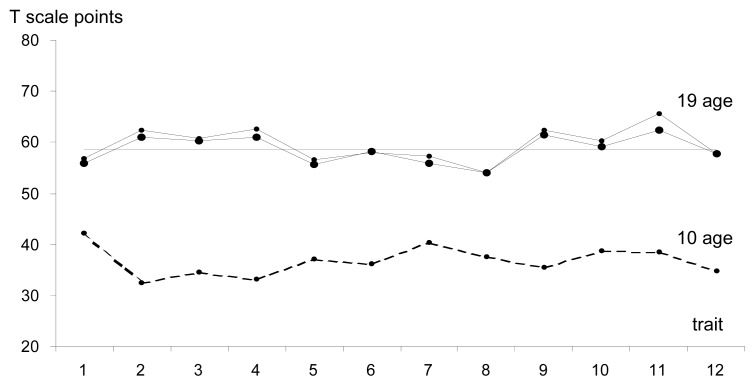
The profile of variables of the morpho-functional model in the adolescent group aged 17–19 in morphological age categories 1. Wrist flexibility, 2. Body height, 3. Shoulder width 4. Length of the arm with a racquet, 5. Selective reaction time, 6. Spatial orientation 7. Coupled motions, 8. Kinaesthetic differentiation, 9. Upper limb MAP 10. Running speed, 11. Lower limb MAP, 12. Abdominal muscle strength

A different structure of the studied model could be observed in the adolescent group where the differentiation of individual variables that comprised the module was the greatest and equalled 0.82 of the standard deviation (Figure 3, Table 1). Among the dominant abilities were MAPs of both lower and upper limbs, while the parameters that scored above average included body height, the length of an arm with a racquet, and shoulder width. Spatial orientation, running speed established on the basis of the zigzag running test and abdominal muscle strength had an average contribution to the sports performance model. Conversely, the effectiveness of a badminton player was determined to a slightly lesser degree by other coordination abilities, such as selective reaction time, coupled motions, kinaesthetic differentiation, and wrist flexibility. In the course of the study, the greatest level of variability in time was displayed by body height and its derivatives, as well as MAP.

A significant role in shaping the level of sports performance in younger teams is initially played by versatile physical fitness and motor coordination in particular. However, in the group of adolescents aged 17–19, the dominant characteristics are mainly parameters relative to MAP development and body height, with the latter concerning predominantly a longer arm with a racquet.

## Discussion

Scientific studies in the field of badminton, especially in Poland, are inconsistent and usually concern advanced players. There are no comprehensive studies in this area. Examinations of children and youth are scarce. Therefore, a comprehensive, multi-faceted approach to identification of morpho-functional factors that determine a sports skill level of young badminton players seems to be a new and original solution of the problem.

As indicated in the introduction, the majority of studies conducted so far have been focused on the effects of anaerobic and aerobic effort on badminton player’s performance. Much less research has focused on human anatomy and the amplitude of motions. Our study revealed that somatic parameters belong to one of the most important groups within the proposed models. At every stage of sports training, the length of an arm with a racquet proved to be an essential prerequisite for badminton. In addition, the results of our own research showed the importance of body height and wrist flexibility. Undoubtedly, the latter characteristic allows badminton players to strike the shuttle in such a way as to give it appropriate power, speed and a flight path. It can be therefore assumed that great flexibility is essential for making hand movements during unconventional strokes that frequently surprise the opponent. It is also important when playing a low serve as well as all combined strokes shortening the shuttle’s flight distance. Other shuttle strokes, and their choice is quite wide, they require in the initial phase of the movement intensive work of the wrist, which only much later is followed by considerable activity of the arm (power). In this sense, the emergence of the described factor is fully logical and understandable. Similarly, the importance of body height has been pointed out by authors of other comparative studies (Chang et al., 2006; Ooi et al., 2009). However, significant differences in the range of this variable exist amongst elite badminton players from Europe, China, Malaysia, Indonesia and Poland (Chang et al., 2006). The studies referenced above also emphasise that body height is not the most important determinant of success in this sports discipline.

The influence of variables determining physical abilities on shaping of the level of sports performance turned out to be considerable. Particular attention needs to be paid, however, to the contribution of the MAP factor, which, in different sampling groups, emerged with various strengths at all levels of sports training. Similar regularities were also observed with regard to cardiorespiratory endurance assessed with the Eurofit running test until volitional exhaustion (1993). This ability was particularly important in the preadolescent and adolescent (14–16 years old) groups. These confirmed facts correspond to the general opinion held by other authors who classify badminton as a speed endurance sports discipline, which mainly results from the nature of the game (Andersen et al., 2007; Hughes and Cosgrovee, 2007; Ooi et al., 2009, Wonisch et al., 2003). The regularities presented above indicate that the positive picture of the game is mainly affected by relatively high development of physical abilities that are certainly reflected in player’s fitness on the court. Furthermore, one should not forget about somatic determinants relative to skilful handling of the racquet. Their composition, what is important, changed with the age of the subjects, primarily with regard to the number of variables and their sequence (hierarchy) in the described morpho-functional models.

Finally, the importance of coordination abilities in the “champion models” needs to be emphasized. As a result of observations being made, a view was formulated that badminton belonged to a group of sports disciplines requiring nonstandard characteristics and a high level of coordination. The vital importance of visual-motor coordination and reaction time in badminton were also proven in comparative studies (Wang et al., 2008, Yuan et al., 1995), whereas the role of these abilities in teaching badminton technique was highlighted by Chin et al. (1995), Mooney and Mutrie (2000) and Sakurai and Ohtsuki (2000). The results of our own research showed that at every stage of sports training, spatial orientation played an essential role. This is quite logical since this ability is important for the assessment of the shuttle’s flight trajectory and the observation of what is happening on the court. However, it needs to be emphasized that the number of coordination variables varied depending on the stage of sports training. In the early stages of badminton training, it was motor coordination that gained considerable dominance, but mainly with respect to spatial orientation, coupled motions and reaction speed to a visual stimulus. In the group of adolescents aged 14–16, the number of coordination components increased to 5. This suggests the necessity to pay particular attention to shaping and mastering the technique during this period of training. It is widely believed that coordination exercises are conducive to acquiring special abilities that, in turn (by means of feedback), increase the level of coordination predispositions. Conversely, in the group of adolescents aged 17–19, the contribution of these abilities slightly decreased. This could result from a smaller dispersion of results, namely, the alignment of their levels in this group of badminton players. However, what also needs to be considered is that these abilities were of a high organisation level (e.g. spatial orientation, selective reaction time).

## Conclusion

The results of the current research allow for the following conclusions:

1. The level of sports performance of the studied badminton players was largely determined by the levels of somatic features, fitness and coordination abilities. At particular stages of training, they created characteristic complexes of variables that did not constitute permanent models.
2. In the early stages of training, the dominance of somatic features and coordination abilities changed for the benefit of speed and strength abilities. It is therefore necessary to adopt appropriate training procedures to reflect these changes.

## Figures and Tables

**Table 1:** The structure of morpho-functional models in the preadolescents aged 11–13, adolescents aged 14–16 and adolescents aged 17–19 in T scale points

Variable	Aged 11–13	Morphological age		Aged 14–16	Morphological age		Aged 17–19	Morphological age	
		10 years old	19 years old		10 years old	19 years old		10 years old	19 years old
Length of the arm with a racquet	39.9	33.2	62.6	52.9	33.2	62.6	60.9	33.2	62.6
Body height				52.9	32.5	62.4	60.9	32.5	62.4
Wrist flexibility	44.8	42.3	56.8				56.0	42.3	56.8
Shoulder width	40.3	34.7	60.8				60.3	34.7	60.8
Agility				50.6	42.2	57.2			
Running speed	42.5	36.2	59.7	52.6	38.8	60.4	59.1	38.8	60.4
Upper limb MAP	40.5	35.6	62.5				61.5	35.6	62.5
Lower limb MAP	40.9	38.6	65.7				62.3	38.6	65.7
Movement frequency				52.4	38	55.5			
Static strength	40.5	38.3	62.5						
Abdominal muscle strength				51.4	34.9	57.7	57.8	34.9	57.7
Cardiorespiratory endurance	43.9	29.7	54.6	53.7	29.7	54.6			
Spatial orientation	40.9	36.3	58.1	54.8	36.3	58.1	58.2	36.3	58.1
Kinaesthetic differentiation				47.0	54.4	50.9	54.1	37.6	54.0
Visual-motor coordination				54.4	28.0	57.4			
Rhythmisation				50.6	46.8	52.0			
Coupled motions	45.4	40.5	57.3				55.8	40.5	57.3
Mean visual reaction time	44.4	33.2	56.9						
Selective reaction time							55.6	37.1	56.6
**Average**	**42.2**			**52.1**			**58.6**		
